# Socioeconomic Moderators of the Association Between Delayed Breastfeeding Initiation and Place of Delivery: Cross-Sectional Study

**DOI:** 10.2196/57254

**Published:** 2024-09-24

**Authors:** Divya Sharma, Jyoti Yadav, Madhu Gupta, Pritam Halder, Abin K Rajan, Tanvi Kiran

**Affiliations:** 1 Department of Community Medicine and School of Public Health Post Graduate Institute of Medical Education and Research (PGIMER) Chandigarh India; 2 Department of Community and Family Medicine AIl India Institute of Medial Science (AIIMS) Gorakhpur India

**Keywords:** breastfeeding, institutional deliveries, delayed initiation, moderation analysis, Indian mothers, socio-economic, cross-sectional study, infant, infancy, infant feeding, human milk, breastfeeding initiation, mother, women, India, healthcare services, awareness, pregnancy, public health

## Abstract

**Background:**

Breastfeeding is a crucial and irreplaceable method of feeding infants. Despite the well-established advantages of early breastfeeding initiation, its progress remains constrained. Over half of Indian mothers witness delayed breastfeeding initiation. Various factors have been implicated to influence breastfeeding initiation, with institutional deliveries emerging as a crucial factor among them.

**Objective:**

We tested the hypothesized association between institutional delivery and initiation delays and identified how various socioeconomic variables moderate (weaken, strengthen, or reverse) the association between breastfeeding initiation delays and place of delivery.

**Methods:**

This cross-sectional study analyses data of 106,569 breastfeeding mothers from the NFHS-5 (National Family Health Survey, 2019-21). Missing data were managed by using a complete case analysis approach. The outcome variable was the timing of breastfeeding initiation for the most recent child, with the place of delivery being the explanatory variable. Socioeconomic factors including age, education level, marital status, place of residence, and wealth index were considered moderating variables. Logistic regression–based moderation analysis explored how these variables influence the relationship between breastfeeding initiation delays and place of delivery. Separate binary logistic regression models analyzed the effect of each moderating variable. Statistical analysis was conducted using IBM SPSS Statistics 26.

**Results:**

The highest occurrence of delayed breastfeeding initiation was observed among mothers aged ≥36 years (58.3%), lacking formal education (60.9%), belonging to lower wealth groups (58.1%), residing in rural areas (57.4%), and having home births (64.1%). Results confirmed the primary hypothesis that institutional delivery significantly and negatively affects delayed breastfeeding initiation (odds ratio [OR] 0.705, 95% CI 0.676-0.735, *P*<.001). Age as a moderating variable significantly affected this association (adjusted OR [aOR] 0.757, 95% CI 0.696-1.307, *P*=.02 for the 15-25 age group). Notably, education level (aOR 0.616, 95% CI 0.429-1.930, *P*=.005 for no education and aOR 0.510, 95% CI 0.429-1.772, *P=*.04 for primary education) and poor wealth index (aOR 0.672, 95% CI 0.528-1.432, *P*=.004) as moderating factors significantly strengthened the negative effect of institutional delivery on delayed initiation. Poor mothers and those without education or a lower level of education (primary) when delivering the child at the health institution further reduced the chances of witnessing delayed initiation.

**Conclusions:**

Institutional delivery significantly lowers the likelihood of delayed breastfeeding initiation, and this negative effect is significantly strengthened when uneducated women or lesser-educated women and those with lower wealth deliver their children at the institutional facilities, underscoring the significance of these moderating factors. Developing strategies targeting these socioeconomic moderating factors is crucial. Tailored awareness programs crafted to address the needs of uneducated mothers from economically disadvantaged backgrounds can enhance coverage. Outreach initiatives aimed at promoting health care service use during pregnancy and delivery, as well as raising awareness about breastfeeding practices, are warranted for the adoption and implementation of early breastfeeding initiation.

## Introduction

Breastfeeding is an invaluable infant feeding practice linked to reduced neonatal mortality. The World Health Organization (WHO) advises initiating early breastfeeding, that is, within the initial hour after delivery. If breastfeeding begins more than an hour after delivery, it is referred to as a delayed initiation [1].

Newborns who are breastfed early receive optimal immunological protection from colostrum [2-4], which contains critical antibodies and nutrients, thereby reducing the chances of neonatal mortality [5,6]. Conversely, delayed initiation is linked to elevated risks of severe illnesses, infections, and increased mortality among newborns and children. There is also a dose-response relationship, indicating that the risk of mortality increases with longer delays in breastfeeding initiation beyond the first hour, extending up to the seventh day [1].

Despite the recognized health advantages of early initiation of breastfeeding (EIBF), a significant portion of newborns in numerous countries are not breastfed within the first hour after birth [2]. In India, various global programs, including the Baby-Friendly Hospital Initiative (BFHI), the Global Strategy for Infant and Young Child Feeding, and the national initiative Mother’s Absolute Affection (MAA), have been implemented to encourage early and exclusive breastfeeding [7-10]. Consequently, there have been improvements in various aspects of breastfeeding practices over time, but progress in breastfeeding initiation has been notably limited [1]. In India, the National Family and Health Survey (NFHS) revealed a negligible change in delayed initiation rates from 58.4% in NFHS-4 (2015-2016) to 58.2% in NFHS-5 (2019-21) [11,12].

A range of socioeconomic, personal, and health factors contribute to delayed breastfeeding initiation [2]. Several studies have identified diverse determinants such as maternal age, education, place of delivery, the practice of discarding the initial milk, mode of delivery, and more associated with breastfeeding initiation [1,3,13]. Many of these studies have underscored the significance of institutional deliveries in fostering breastfeeding initiation. Health workers counsel and motivate mothers who give birth in health care institutions to breastfeed within the first hour after delivery, promoting early breastfeeding [1-3,6,13]. The Indian government has also emphasized the significance of institutional deliveries for the holistic well-being of mothers and children. [14]. The theoretical viewpoint points out that delivering the infant in the institutional setting elevates the probability of early breastfeeding initiation [6,15,16]. In other words, institutional delivery reduces the likelihood of delayed initiation of breastfeeding.

This study, using nationally representative data from over 100,000 breastfeeding mothers, tests the hypothesis that institutional delivery negatively affects the delays in breastfeeding initiation, that is, delivering a child in an institution results in a lesser likelihood of delayed breastfeeding among Indian mothers. The study further tests the hypothesis that this association between institutional delivery and delayed initiation of breastfeeding is significantly influenced (moderated) by socioeconomic variables; that is, the study investigates whether socioeconomic variables weaken, strengthen, or reverse the aforementioned theorized association between institutional delivery and initiation delays. To our knowledge, such moderation analysis has been under researched in the Indian context. The findings will help identify factors that limit the effectiveness of institutional delivery in promoting early breastfeeding, which will help facilitate timely interventions to improve maternal and child health outcomes and reduce neonatal and child mortality in India.

## Methods

### Study Data

The fifth in the series, NFHS-5, was conducted by the International Institute for Population Sciences (IIPS) in Mumbai under the guidance of the Ministry of Health and Family Welfare (MoHFW), Government of India. It furnishes comprehensive data on demographics, health, and nutrition for India and its individual states, union territories, and 707 districts as of March 31, 2017[12]. To acquire access to the cross-sectional data from NFHS, the registration was done on the DHS (Demographic and Health Surveys) program website [17], and the data was extracted subsequently.

### Study Setting and Participants

The NFHS-5 survey gathered data from a wide-ranging sample, including 636,699 households, 724,115 women, and 101,839 men, spanning 707 districts, 28 states, and 8 union territories. A consistent sample design was used to ensure accurate national, state, or union territory and district representation. Each district was further categorized into urban and rural areas. The NFHS-5 survey used a stratified 2-stage sampling approach, using the 2011 census data to select Primary Sampling Units (PSUs), with villages in rural areas and Census Enumeration Blocks (CEBs) in urban areas serving as these units. The detailed study design and sampling strategy are mentioned in the NFHS-5 survey report [12]. The survey used 4 distinct schedules or questionnaires (Household, woman, man, and biomarker) and was conducted in 18 local languages, aided by computer-assisted personal interviewing (CAPI). This study specifically included 106,569 breastfeeding mothers in its analysis [12].

### Study Variables

#### Outcome and Explanatory Variables

The variable of interest (outcome) was the timing of breastfeeding initiation of the last-born child, assessed with question Q499C: “How long after birth did you start breastfeeding (Name)?” The participants’ responses were categorized into 2 groups: early initiation (within 1 hour) and delayed initiation (more than 1 hour) [3,6]. Based on this, we labeled the variable of interest (outcome or dependent variable) as “initiation delays” (presence and absence). The place of delivery was chosen as the explanatory (independent) variable, assessed with question Q448: “Where did you give birth to (Name)?” The place of delivery was dichotomized into “home” and “institutional delivery.” “Institutional” delivery was coded as 1 and “home” as the reference category in the regression analysis framework, which is explained in the statistical section below. The presence of a significant positive association of early breastfeeding initiation with institutional delivery, as reflected in the literary and academic works in the Indian context, was the impelling factor for taking up the aforementioned as the outcome and explanatory variables, respectively [18,19]. In this study, the effect of place of delivery on initiation delays (main association) was examined through the following baseline (unadjusted) logistic regression model, against which all moderating effects are then compared:



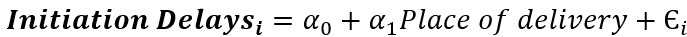



Where, *α*_0_ denotes the regression intercept term, and *α*_1_ denotes the regression slope coefficient; Є*_i_* denotes the error term.

#### Moderating Variables

The analysis included various socioeconomic factors as moderating variables. These factors covered age, level of education, marital status, place of residence, and wealth index. Age was grouped into 3 categories: 15-25, 26-35, and 36 years and older. Educational attainment was classified into 4 levels: no education, primary, secondary, and higher education. Marital status was categorized into 2 groups: married and divorced, separated, unmarried, or widowed. The wealth index was divided into 3 categories: poor, middle, and rich. The place of residence was categorized into urban and rural areas based on geographical location.

### Final Included Sample and Missing Data Analysis

**Figure 1:**
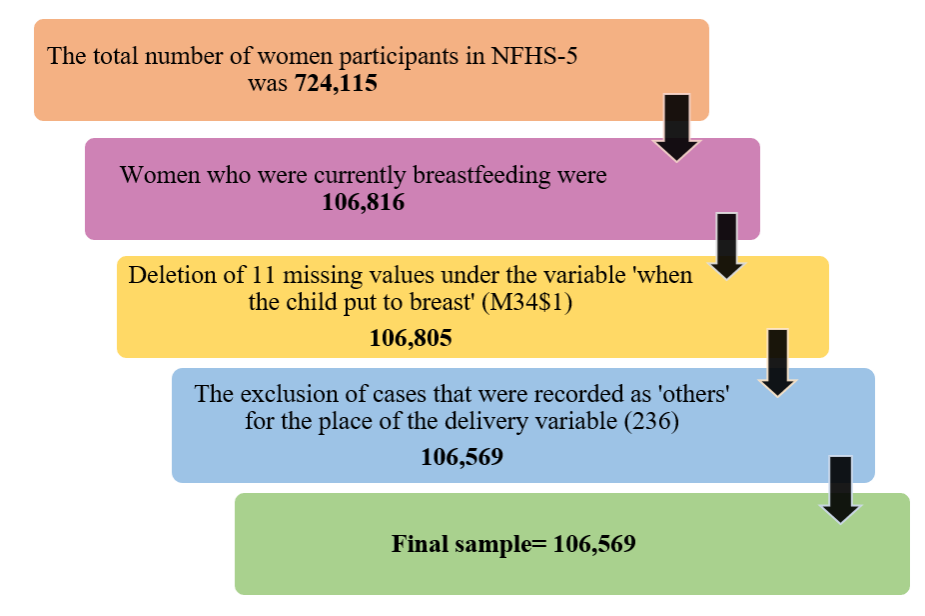
Flowchart showing participants’ selection process in this study. NFHS-5: National Family Health Survey, 2019-21.

We examined and excluded missing data for all variables of interest, including both outcome and explanatory variables, through a complete case analysis approach [20]. Following the removal of missing values in the dependent variable and the exclusion of cases recorded as “others” in the place of the delivery variable, the total number of participants included in the study amounted to 106,569. The participant selection process is presented in Figure 1.

### Sample Weights

Given the nonproportional allocation of the sample across various survey domains and their urban and rural areas, the use of sampling weights is essential for any analysis using the survey data. Consequently, all analyses were weighted using NFHS-5 sample weights [21].

### Moderation Analysis

**Figure 2:**
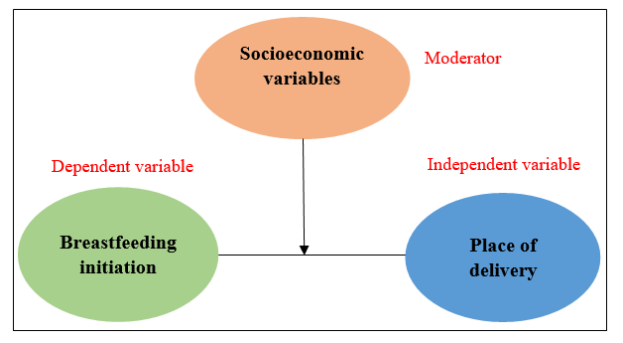
Theoretical model for moderation analysis.

Moderation refers to a scenario in which the association between 2 variables is not constant but somewhat influenced by the values of a third variable known as a moderator variable. The initial conceptual framework for moderation analysis was provided by Baron and Kenny [22]. A moderator variable can exert 3 possible effects on the relationship between independent and dependent variables. Particularly, a moderator can (1) weaken the relationship, (2) strengthen the relationship, or (3) reverse the relationship [23]. Thus, this moderator variable, or construct, has the ability to alter the strength or even the direction of the relationship between the 2 entities within a model [24]. In moderation analysis, first, the direct effect of a moderator variable on the outcome or dependent variable was estimated using the unadjusted binary logistic regression model framework, followed by the moderating effect, which is studied by including the interaction term between the independent and moderator variables using the adjusted logistic regression modeling [25,26]. In this study, moderation analysis was used to delve deeper into the moderating effect of the socioeconomic variables on the strength of the relationship between the initiation of breastfeeding and the place of delivery (main association; Figure 2).

Separate binary logistic regression models were fitted to study the effect of each one of the moderating variables. The statistical analysis was performed using IBM SPSS Statistics 26.

The regression models can be expressed through the following equations:

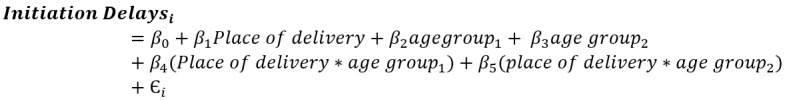

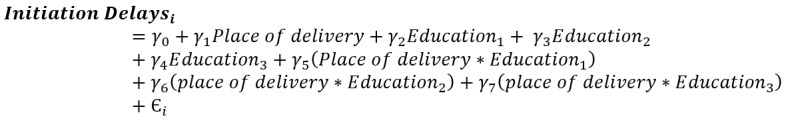

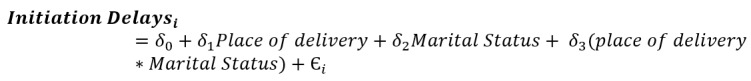

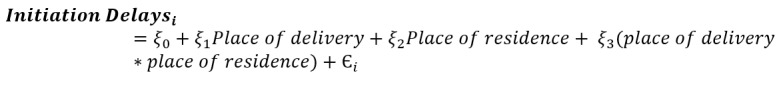

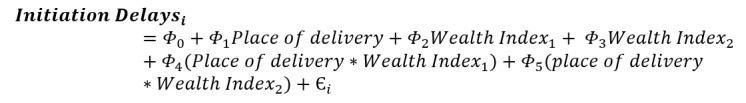











Where 
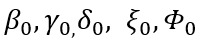
 denotes the regression intercept terms, 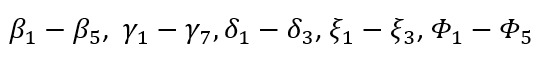 denotes the regression slope coefficients, and Є*_i_* denotes the error term.

### Ethical Considerations

This study used preexisting secondary data with no identifiable information extracted from the NFHS-5 study. The DHS Program’s website hosts NFHS-5, an anonymous data set freely available to the public upon reasonable data request, and does not allow for the identification of survey respondents. The original NFHS-5 study adhered to research ethics, with ethical approval from the IIPS, Mumbai, for NFHS-5 (2019-21). In addition, the informed consent form and International Review Board (IRB) also reviewed the survey and granted ethical approval. Respondents granted their signed consent after receiving complete information about the survey’s purpose and procedures. The interviews were conducted after obtaining explicit consent from each participant [27].

## Results

### Socioeconomic Characteristics of the Study Population

The study included 106,569 breastfeeding mothers and Table 1 provides an overview of their sociodemographic characteristics. Among mothers aged 15-25 years and those aged 26-35 years, the rates of delayed initiation were quite similar at 56.3% (28,963/51,442) and 56.6% (27,121/47,912), respectively. In the 36 years and above age group, the delayed initiation rate was reported to be 58.3% (2805/4812). Notably, a majority of mothers (60.9%, 12,804/21,023) without formal education experienced delayed breastfeeding initiation, while more than half of those with higher education (57.6%, 9512/16,501) practiced delayed initiation. The study documented that 56.5% (58,363/103,215) of married women and 55.2% (525/951) of divorced, separated, widowed, or unmarried women delayed the initiation of breastfeeding.

**Table 1:** Socioeconomic characteristicsa of breastfeeding mothers in India: descriptive measures using cross-sectional survey weights from NFHS-5 (National Family Health Survey, 2019-21; weighted estimates).

Variables		Mothers initiating early breastfeeding, n/N (%)	Mothers initiating delayed breastfeeding, n/N (%)
**Age (years)**			
	15-25	22,479/51,442 (43.7)	28,963/51,442 (56.3)
	26-35	20791/47,912 (43.4)	27,121/47,912 (56.6)
	36 and above	2007/4812 (41.7)	2805/4812 (58.3)
**Highest education level**			
	No education	8219/21,023 (39.1)	12,804/21,023 (60.9)
	Primary	5502/12,571 (43.8)	7069/12,571 (56.2)
	Secondary	24,569/54,073 (45.4)	29,504/54,073 (54.6)
	Higher education	6989/16,501 (42.4)	9512/16,501 (57.6)
**Current marital status**			
	Married	44,852/103,215 (43.5)	58,363/103,215 (56.5)
	Divorced, separated, unmarried, or widowed	426/951 (44.8)	525/951 (55.2)
**Wealth index**			
	Poor	21,299/50,788(41.9)	29,489/50,788 (58.1)
	Middle	8970/20,243 (44.3)	11,273/20,243 (55.7)
	Rich	15,009/33,135 (45.3)	18,126/33,135 (54.7)
**Type of place of residence**			
	Urban	11,839/25,643 (46.2)	13,804/25,643 (53.8)
	Rural	33,439/78,524 (42.6)	45,085/78,524 (57.4)
**Place of delivery**			
	Home	3688/10,269 (35.9)	6581/10,269 (64.1)
	Institutional delivery	41,590/93,898 (44.3)	52,308/93,898 (55.7)

Mothers with a “poor” wealth index displayed elevated rates of delayed initiation, with 58.1% (29,489/50,788) experiencing delays. Those in the middle and rich wealth index categories recorded 55.7% (11,273/20,243) and 54.7% (18,126/33,135), respectively. Rural-dwelling mothers had a higher rate of delayed initiation at 57.4% (45,085/78,524). Among mothers who delivered in institutional settings, 55.7% (52,308/93,898) reported delayed initiation, while those delivering at home showed a higher prevalence of delays at 64.1% (6581/10,269).

### Strength of Association Between Initiation Delays and Place of Delivery

The primary outcome of our study, that is, delays in breastfeeding initiation, is significantly and negatively associated with institutional delivery. The odds of delayed initiation (odds ratio [OR] 0.705, 95% CI 0.676-0.735, *P*<.001) were significantly lower for the mothers who delivered babies at the institutional facility than for those who delivered their child at home. This confirms the hypothesis that institutional delivery results in significantly lesser odds (29.5%) for delayed breastfeeding among Indian mothers.

### Direct Effects of Moderating Variables on Delayed Initiation

Table 2 presents the direct effects of moderating variables on delayed initiation among Indian breastfeeding mothers using binary logistic regression modeling. Mothers aged 15-25 years (OR 0.922, 95% CI 0.868-0.979, *P*=.008) and those aged 26-35 years (OR 0.933, 95% CI 0.879-0.991, *P*=.02) documented slightly lower odds of delayed initiation of breastfeeding compared with those aged 36 years and older. The odds of delayed initiation were highest among mothers with no education (OR 1.145, 95% CI 1.098-1.193, *P*<.001) compared with those with higher education. However, these odds significantly decreased with higher education levels, namely primary (OR 0.944, 95% CI 0.901-0.989, *P*=.01) and secondary education levels (OR 0.882, 95% CI 0.852-0.914, *P*<.001). The findings suggested a significant association between the wealth index and delayed breastfeeding initiation. Mothers in the poor category are more likely to practice delayed initiation than those in the rich category, as evident by significantly higher odds (OR 1.146, 95% CI 1.115-1.179, *P*<.001). Mothers in the middle wealth index category also demonstrated significantly higher odds than those in the rich category for delayed initiation of breastfeeding (OR 1.041, 95% CI 1.005-1.078, *P*=.02). Mothers residing in rural areas displayed significantly higher odds of delayed initiation (OR 1.156, 95% CI 1.124-1.190, *P*<.001) than those in urban areas. The classification accuracy for each of the moderating variables is above the threshold of 0.5.

**Table 2:** Direct effects of moderating variables on delayed initiation among Indian breastfeeding mothers: results of unadjusted binary logistic regression models (weighted estimates).

Variables		OR^a^ (95 % CI)	*P* value
**Age (years)**			
	15-25	0.922^b^ (0.868-0.979)	.008
	26-35	0.933^c^ (0.879-0.991)	.02
	≥36	Reference	—^d^
**Highest education level**			
	No education	1.145^e^ (1.098-1.193)	<.001
	Primary	0.944^c^ (0.901-0.989)	.01
	Secondary	0.882^e^ (0.852-0.914)	<.001
	Higher education	Reference	—
**Current marital status**			
	Married	Reference	—
	Divorced, separated, unmarried, or widowed	0.948 (0.834-1.078)	.41
**Wealth index**			
	Poor	1.146^e^ (1.115-1.179)	<.001
	Middle	1.041^c^ (1.005-1.078)	.02
	Rich	Reference	—
**Type of place of residence**			
	Urban	Reference	—
	Rural	1.156^e^ (1.124-1.190)	<.001

^a^OR: odds ratio.

^b^Significant at *P*≤.01.

^c^Significant at *P*≤.05.

^d^Not applicable.

^e^Significant at *P*≤.001.

### Moderating Effect on the Strength of the Relationship Between Delayed Breastfeeding Initiation and Place of Delivery

Separate multivariable logistic regression models with interactive terms for moderation analyses were fitted (Table 3). The detailed regression models and the procedure of estimation of moderation effects are represented in (Table S1 in Multimedia Appendix 1). The moderation analysis findings indicated that among mothers in the 15-25 years age group who delivered in institutions, the odds of delayed breastfeeding initiation were 24.3% lower (estimated moderating effect in terms of adjusted OR [aOR] 0.757, 95% CI 0.696-1.307, *P*=.02) as compared with women of higher age groups and those delivering the child at home. These odds were 29.5% lower (OR 0.705, 95% CI 0.676-0.735, *P*<.001) when women delivered in an institution in the absence of this moderating variable, thereby indicating that the inclusion of age as a moderating variable weakened the effect of institutional delivery on delayed initiation (main association).

Interestingly, education as a moderating variable has significantly strengthened the negative effect of institutional delivery on delayed initiation. It was observed that after delivering in health institutions, mothers with no education had approximately 38% lesser odds of delayed initiation (estimated moderating effect in terms of aOR 0.616, 95% CI 0.429-1.930, *P*=.005). The odds of delayed initiation were 29.5% lower (OR 0.705, 95% CI 0.676-0.735, *P*<.001) when women delivered in the institution in the absence of education as a moderating variable. Similarly, mothers with primary education who delivered in institutions exhibited 49% lower odds (aOR 0.510, 95% CI 0.429-1.772, *P*=.04) than women with higher education and those delivering the child at home. These odds were lower (49%) as compared when women delivered in an institution, in the absence of moderating effect of education (OR 0.705, 95% CI 0.676-0.735, *P*<.001), thereby indicating that the mothers with no education and lower level of education (primary), when delivered the child at the health institution have further reduced chances of having delayed initiation.

In addition, mothers from poor socioeconomic status (wealth index) who delivered in the institutions showed approximately 33% lower odds (estimated moderating effect in terms of aOR 0.672, 95% CI 0.528-1.432, *P*=.004) as compared with those with rich socioeconomic backgrounds. These odds were lower (33%) as compared with when women delivered in an institution in the absence of wealth index as a moderating variable (29.5% lower odds with OR 0.705, 95% CI 0.676-0.735, *P*<.001), thereby indicating that the mothers from poor wealth index when delivered the child at the health institution have even lesser chances of having delayed initiation. This substantiates that the wealth index as a moderating variable has strengthened the negative effect of institutional delivery on delayed initiation (main association).

All the regression models involving moderation analysis displayed a “good fit.” The classification accuracy of these models in predicting the outcome (Table 3) was adequate and above the requisite threshold of 50% [28,29]. The omnibus test of model coefficients was also statistically significant, indicating that the model (with explanatory variables) has a good fit.

**Table 3:** Moderating effect of socioeconomic variables on the association between delayed initiation of breastfeeding and place of delivery: results of multivariable logistic regression models (weighted estimates). Classification accuracy for all models >0.5; Omnibus Tests of Model Coefficients *P*<.001.

Variables				aOR^a^		*P* value		Estimation of moderating effects		*P* value			Interpretation		
Baseline model (unadjusted): effect of institutional delivery on initiation delays															Institutional delivery lowers the likelihood of delayed inhiation (main association)
	**Place of delivery**														
		Home	Reference		—		—^b^		—						
		Institution	0.705^c,d^		<.001		—		—						
Model 1: moderating effect of age on the association between initiation delays and institutional delivery															Age of the breastfeeding women significantly moderates the main association
	**Place of delivery**														
		Home	Reference		—		—		—						
		Institution	0.821^e^		.02		—		—						
	**Age (years)**														
		15-25	1.121		.14		—		—						
		26-35	1.065		.41		—		—						
		36 and above	Reference		—		—		—						
	**Place of delivery×age (years)**														
		Institution×15-25	0.823^e^		.02		0.821 × 1.121 × 0.823 = 0.757^e^		.02			The main association is significantly weakened for women aged 15-25 years			
		Institution×26-35	0.876		.11		0.821 × 1.065 × 0.876 = 0.765		.11			No significant effect			
		Home×36 and above	Reference		—		—		—			—			
Model 2: moderating effect of level of education on the association between initiation delays and institutional delivery															Education level of breastfeeding women significantly moderates the main association
	**Place of delivery**														
		Home	Reference		—		—		—						
		Institution	0.557^d^		<.001		—		—						
	**Highest level of education**														
		No education	0.750^e^		.03		—		—						
		Primary	0.685^f^		.007		—		—						
		Secondary	0.729^e^		.02		—		—						
		Higher education	Reference		—		—		—						
	**Place of delivery×highest level of education**														
		Institution×no education	1.474^f^		.005		0.557 × 0.750 × 1.474 = 0.616^f^		.005			The main association is significantly strengthened for women without education			
		Institution×primary	1.337^e^		.04		0.557 × 0.685 × 1.337 = 0.510^e^		.04			The main association is significantly strengthened for women with primary education			
		Institution×secondary	1.190		.20		0.557 × 0.729 × 1.190 = 0.483		.20			No significant effect			
		Home×higher education	Reference		—		—		—			—			
Model 3: moderating effect of marital status on the association between initiation delays and institutional delivery															
	**Place of delivery**														
		Home	Reference		—		—		—			—			
		Institution	0.703^d^		<.001		—		—			—			
	**Marital status**														
		Married	0.784		.54		—		—			—			
		Divorced, separated, unmarried, or widowed	Reference		—		—		—			—			
	**Place of delivery×current marital status**														
		Institution×married	1.227		.29		0.703 × 0.784 × 1.227 = 0.676		.29			No significant effect			
		Home×divorced, separated, unmarried, or widowed	Reference		—		—		—			—			
Model 4: moderating effect of place of residence on the association between initiation delays and institutional delivery															
	**Place of delivery**														
		Home	Reference		—		—		—			—			
		Institution	0.755^d^		<.001		—		—			—			
	**Place of residence**														
		Rural	1.203^f^		.002		—		—			—			
		Urban	Reference		—		—		—			—			
	**Place of delivery×place of residence**														
		Institution×rural	0.939		.30		0.755 × 1.203 × 0.939 = 0.852		.30			No significant effect			
		Home×urban	Reference		—		—		—			—			
Model 5: moderating effect of wealth index on the association between initiation delays and institutional delivery															
	**Place of delivery**														
		Home	Reference		—		—		—			Wealth Index significantly moderates the main association			
		Institution	0.605^d^		<.001		—		—			—			
	**Wealth index**														
		Poor	0.897		.13		—		—			—			
		Middle	0.898		.23		—		—			—			
		Rich	Reference		—		—		—			—			
	**Place of delivery×wealth index**														
		Institution×poor	1.238^f^		.004		0.605 × 0.897 × 1.238 = 0.672^f^		.004			The main association is significantly strengthened for women with poor wealth index			
		Institution×middle	1.150		.13		0.605 × 0.898 × 1.150 = 0.625		.13			No significant effect			
		Home×rich	Reference		—		—		—			—			

^a^aOR: adjusted odds ratio.

^b^Not applicable.

^c^Unadjusted odds ratios.

^d^Significant at *P*≤.001.

^e^Significant at *P*≤.05.

^f^Significant at *P*≤.01.

## Discussion

### Principal Findings

In this research endeavor, we examined the association between institutional childbirth and the delayed initiation of breastfeeding using data sourced from NFHS-5. The moderating influence of diverse socioeconomic parameters on the strength of the relationship between breastfeeding initiation and institutional childbirth was investigated. The primary aim was to identify impediments impacting the timely breastfeeding initiation within institutional childbirth scenarios, with the overarching objective of ameliorating maternal and child health outcomes within the context of India.

The study on 106,569 mothers highlights the prevalence of delayed breastfeeding initiation. Notably, delayed initiation rates were similar between mothers aged 15-25 years and those aged 26-35 years, suggesting similar contributing factors. However, a slight increase was observed in mothers aged 36 years and older, indicating unique challenges for older mothers, such as health issues, complications, previous breastfeeding experiences, societal perceptions, and support systems. Binary logistic regression modeling showed that mothers aged 15-25 years and those aged 26-35 years had slightly lower odds of delayed initiation compared with those aged 36 years and older. A study by Kitano et al [30] among Japanese mothers also found that first-time older mothers faced higher risks of delayed exclusive breastfeeding initiation. Results from a secondary analysis based on the WHO Global survey suggested that maternal age, specifically 35 years or older, is associated with a decreased likelihood of initiating breastfeeding within 1 hour after birth. However, this association becomes nonsignificant after adjusting for potential confounding factors [2].

The majority of mothers without formal education experienced delayed breastfeeding initiation, which might be due to a lack of awareness or access to proper health care information among this demographic. The direct effects of moderating variables indicated that the mothers with no formal education demonstrated significantly higher odds of delayed initiation relative to their counterparts with higher educational attainment. Along similar lines, a study showed that among mothers without formal education, the proportion of early initiation of breastfeeding and exclusive breastfeeding was the smallest [31].

The study’s documentation of delayed breastfeeding initiation indicates that marital status alone may not be a significant determinant of breastfeeding behavior. Furthermore, the mothers of the poor wealth index category exhibited a heightened prevalence of delayed breastfeeding initiation relative to counterparts categorized under middle and high wealth index strata. The regression analysis (direct effects of moderating variables) also underscores a noteworthy association between wealth index and delayed breastfeeding initiation. Mothers categorized as economically disadvantaged (poor) exhibited markedly higher odds of delayed initiation compared with those classified as rich [19]. Furthermore, mothers within the middle wealth index category also displayed significantly elevated odds of delayed initiation compared with their affluent counterparts. Mothers residing in rural areas demonstrated notably higher odds of delayed initiation than those dwelling in urban locales. Similar results were shown in another study in Ethiopia, where mothers residing in urban areas were 29% more inclined to initiate early breastfeeding than those living in rural areas [32].

Mothers who gave birth outside of a health care facility were more likely to have increased odds of delayed initiation [31]. Our research also showed that the mothers opting for home delivery exhibited a greater prevalence of delays. The principal hypothesis (main association) proving the finding of our investigation was that the delay of breastfeeding initiation exhibited a statistically significant negative association with institutional delivery. These results are consistent with the findings of other studies that show that mothers delivering in health care facilities exhibited a reduced likelihood of encountering delayed onset of breastfeeding [1,33]. Along similar lines, a greater incidence of timely breastfeeding initiation was identified among mothers in public health care institutions than those in private facilities or at home [32,34].

The results of the regression-based moderation analyses in our study provided valuable insights into how certain variables interact with the relationship between institutional delivery and delayed breastfeeding initiation. While studying age as a moderating variable, it came to light that for younger mothers, delivering in institutions appears to have a mitigating effect on delayed initiation. The negative effect of institutional delivery on delayed initiation is significantly strengthened when considering education. It was observed that the mothers with no education and a lower level of education (primary), when delivering the child at the health institution, witness further reduced chances of having delayed initiation. This indicates that the impact of institutional delivery on reducing initiation delays is more pronounced for mothers without formal education or lower levels of education, which highlights the importance of breastfeeding and nutritional counseling provided by midwives and obstetricians in health institutions at the time of childbirth [35].

Similar to education, the wealth index as a moderating variable has strengthened the negative effect of institutional delivery on delayed initiation (main association). Mothers from poor wealth index, when delivering the child at a health institution, witness even lesser chances of having delayed breastfeeding initiation. This might be because factors such as poverty-related factors including lack of awareness, knowledge, support, and other psychosocial factors get compensated to some extent when poor women delivering at the health institution gets continuum of antenatal and postnatal care and breastfeeding support services under programs such as MAA [9,35]. Considering the complex moderating effects and interplay of socioeconomic factors, these insights can inform targeted interventions to improve breastfeeding practices among vulnerable populations.

### Strengths

This study’s merits lie in its use of a nationally representative survey with a substantial sample size, affording sufficient power to incorporate diverse potential factors into the analysis. Furthermore, this study ensured greater reliability of its results by applying appropriate statistical adjustments, including using sample weights. This study distinguishes itself through its novel methodology for examining how different socioeconomic factors moderate the strength of association between institutional delivery and breastfeeding initiation among Indian mothers. The study highlights the importance of considering moderating variables such as age, education, and wealth when examining the relationship between institutional delivery and breastfeeding initiation timing. The findings suggest that while institutional delivery results in a reduced probability of delayed breastfeeding initiation for specific demographic groups (such as younger mothers), this effect can be strengthened by education levels and socioeconomic status. The study, thus, offers valuable insights for tailoring interventions to address specific moderating factors associated with delayed initiation and develop effective management strategies by identifying areas needing attention.

### Limitations

This study faces a few limitations. The data set’s cross-sectional design precludes any causal inference. As the study relies on data sourced from nationally representative surveys, certain crucial variables could not be incorporated due to data gaps or missing information. Due to the self-reporting nature, asking mothers about breastfeeding initiation introduces the possibility of recall bias, potentially resulting in either underestimation or overestimation of delayed initiation. Nevertheless, this study minimized recall bias by focusing the analysis on currently breastfeeding mothers and their last-born children.

### Conclusion

The research investigated the moderating effects of socioeconomic variables on the association between institutional childbirth and delayed breastfeeding initiation. Given the positive influence of institutional delivery on breastfeeding initiation, it is imperative to enhance the promotion of institutional deliveries through national programs to attain full coverage. The direct unadjusted effects of socioeconomic factors such as age, education level, wealth index, and place of residence play a crucial role in breastfeeding initiation. Mothers of the advanced age group (36 years and older) who lack formal education, belong to low-income households and reside in rural areas are at a higher likelihood of delaying breastfeeding initiation. Institutional delivery significantly lowers the likelihood of delayed breastfeeding initiation, and this negative effect is significantly strengthened when uneducated or less educated women with poor wealth status deliver their children to institutional facilities, underscoring the significance of these moderating factors.

Developing strategies targeting these socioeconomic factors is crucial. Tailored awareness programs designed explicitly for mothers lacking formal education can enhance coverage. Interventions should be crafted to address the needs of rural and uneducated mothers from economically disadvantaged backgrounds. Outreach initiatives aimed at promoting health care service use during pregnancy and delivery, as well as raising awareness about breastfeeding practices, are essential measures to improve the adoption and implementation of early breastfeeding initiation.

In conclusion, our study provides valuable insights into the complex interplay of socioeconomic factors affecting breastfeeding initiation within institutional childbirth settings in India. The moderating factors that weaken and strengthen the association between delayed initiation and institutional, when identified, can guide early interventions aimed at enhancing breastfeeding practices at the health facility level to achieve overall maternal and child health, especially for low- and middle-income countries such as India.

## Appendix

Multimedia Appendix 1 Detailed Estimation of Moderating effect of socio-economic variables on the association between delayed initiation of breastfeeding and place of delivery.

## Abbreviations

aOR: adjusted odds ratio
BFHI: Baby-Friendly Hospital Initiative
CAPI: computer-assisted personal interviewing
CEB: census enumeration block
DHS: Demographic and Health Surveys
EIBF: early initiation of breastfeeding
IIPS: International Institute for Population Sciences
IRB: international review board
MAA: Mother’s Absolute Affection
MoHFW: Ministry of Health and Family Welfare
NFHS: National Family Health Survey
OR: odds ratio
PSU: primary sampling unit
WHO: World Health Organization

## Data Availability

The study uses secondary data, which is publicly accessible and available from the DHS program website. The data sets analyzed during this study are available in the Mendeley Data repository [36].
