# Supplementary material for: Socioeconomic Moderators of the Association Between Delayed Breastfeeding Initiation and Place of Delivery: Cross-Sectional Study
Source: JMIR Public Health Surveill. 2024 Sep 24;10:e57254. doi: 10.2196/57254 (PMC11462097; doi:10.2196/57254)
Supplement: Multimedia Appendix 1 [file publichealth_v10i1e57254_app1.docx]

**Supplementary Table 1: Detailed Estimation of Moderating effect of socio-economic variables on the association between delayed initiation of breastfeeding and place of delivery**

| **Variables** | **Categories** | **aOR** | ***P*- values** | **Detailed Estimation of  Moderating Effects** |
| --- | --- | --- | --- | --- |
| ${\boldsymbol{Baseline Model-}Initiation Delays}_{\boldsymbol{i}}=\alpha_{0}+\alpha_{1}Place of delivery$ +$Є_{i}$ | | | | |
| **Place of delivery** | Home | Ref. | --- | --- |
|  | Institution | 0.705***^†^ | 0.00 |  |
| ***Model 1-*** ${Initiation Delays}_{i}=\beta_{0}+\beta_{1}Place of delivery+\beta_{2}{agegroup}_{1}+ \beta_{3}{age group}_{2}+\beta_{4}(Place of delivery*{age group}_{1})+\beta_{5}(place of delivery*{age group}_{2})+Є_{i}$ | | | | |
| **Place of delivery** | Home | Ref. | --- | Multiply the adjusted odds ratios corresponding to category ‘institution’ with that of specific age category and its corresponding interaction term. |
|  | Institution | 0.821* | 0.01 |  |
| **Age** | 15-25 | 1.121 | 0.14 |  |
|  | 26-35 | 1.065 | 0.41 |  |
|  | 36 and above | Ref. | --- |  |
| **Place of delivery**×**Age**  **(Interaction/moderation terms)** | Institution ×15-25 | 0.823* | 0.021 | 0.821×1.121×0.823 = **0.757*** |
|  | Institution × 26-35 | 0.876 | 0.11 | 0.821×1.065×0.876 = 0.765 |
|  | Home×36 and above | Ref. | --- |  |
| **Model 2-** ${Initiation Delays}_{i}=\gamma_{0}+\gamma_{1}Place of delivery+\gamma_{2}{Education}_{1}+ \gamma_{3}E{ducation}_{2}+{\gamma_{4}E{ducation}_{3}+\gamma}_{5}\left( Place of delivery*{Education}_{1} \right)+\gamma_{6}\left( place of delivery*E{ducation}_{2} \right)+\gamma_{7}\left( place of delivery*E{ducation}_{3} \right)+Є_{i}$ | | | | |
| **Place of delivery** | Home | Ref. | --- | Multiply the adjusted odds ratios  corresponding to category ‘institution’ with that of ‘specific level of education’ category and its corresponding  interaction term |
|  | Institution | 0.557*** | 0.00 |  |
| **Highest level of education** | No education | 0.750* | 0.03 |  |
|  | Primary | 0.685** | 0.007 |  |
|  | Secondary | 0.729* | 0.02 |  |
|  | Higher education | Ref. | --- |  |
| **Place of delivery**×**Highest level of education**  **(Interaction/moderation terms)** | Institution×No education | 1.474** | 0.005 | 0.557×0.750×1.474 = **0.616**** |
|  | Institution×Primary | 1.337* | 0.04 | 0.557×0.685×1.337 = **0.510*** |
|  | Institution×Secondary | 1.190 | 0.20 | 0.557×0.729×1.190 = 0.483 |
|  | Home×Higher education | Ref. | --- |  |
| **Model 3-** ${Initiation Delays}_{i}=\delta_{0}+\delta_{1}Place of delivery+\delta_{2}Marital Status+ \delta_{3}(place of delivery*Marital Status)+Є_{i}$ | | | | |
| **Place of delivery** | Home | Ref. | --- | Multiply the adjusted odds ratios corresponding to category ‘institution’  with that of ‘married category’ and its  corresponding interaction term |
|  | Institution | 0.703*** | 0.00 |  |
| **Marital status** | Married | 0.784 | 0.18 |  |
|  | Divorced/separated/ unmarried/widowed | Ref. | --- |  |
| **Place of delivery**×**Current marital status**  **(Interaction/moderation term)** | Institution×Married | 1.227 | 0.29 | 0.703×0.784×1.227 = 0.676 |
|  | Home×Divorced/separated/ unmarried/widowed | Ref. | --- |  |
| **Model 4** - ${Initiation Delays}_{i}=\xi_{0}+\xi_{1}Place of delivery+\xi_{2}Place of residence+ \xi_{3}(place of delivery*place of residence)+Є_{i}$ | | | | |
| **Place of delivery** | Home | Ref. | --- | Multiply the adjusted odds ratios  corresponding to category ‘institution’ with that of ‘rural category’ and its  corresponding interaction term |
|  | Institution | 0.755*** | 0.00 |  |
| **Place of residence** | Rural | 1.203** | 0.002 |  |
|  | Urban | Ref. | --- |  |
| **Place of delivery**×**Place of residence**  **(Interaction/moderation term)** | Institution×Rural | 0.939 | 0.30 | 0.755×1.203×0.939 = 0.852 |
|  | Home×Urban | Ref. | --- |  |
| **Model 5-** $\boldsymbol{Initiation Delays}_{\boldsymbol{i}}=\Phi_{0}+\Phi_{1}Place of delivery+\Phi_{2}{Wealth Index}_{1}+ \Phi_{3}{Wealth Index}_{2}+\Phi_{4}(Place of delivery*{Wealth Index}_{1})+\Phi_{5}(place of delivery*{Wealth Index}_{2})+Є_{i}$ | | | | |
| **Place of delivery** | Home | Ref. | --- | Multiply the adjusted odds ratios  corresponding to category ‘institution’ with that of ‘specific wealth index category’ and its corresponding  interaction term |
|  | Institution | 0.605*** | 0.00 |  |
| **Wealth Index** | Poor | 0.897 | 0.13 |  |
|  | Middle | 0.898 | 0.23 |  |
|  | Rich | Ref. | --- |  |
| **Place of delivery**×**Wealth index**  **(Interaction/moderation term)** | Institution×Poor | 1.238** | 0.004 | 0.605×0.897×1.238 = **0.672**** |
|  | Institution×Middle | 1.150 | 0.13 | 0.605×0.898×1.150 = 0.625 |
|  | Home×Rich | Ref. | --- |  |

Note: - Asterisks denote significant *P* values.

* *P* value <.05

** *P* value <.01

*** *P* value <.001
